# Radiation Exposure in Minimally Invasive Cervical Spine Surgery: A Systematic Review

**DOI:** 10.3390/medicina62050977

**Published:** 2026-05-17

**Authors:** Dong Hun Kim, Jung-Woo Hur, Jae Taek Hong

**Affiliations:** 1 Department of Neurosurgery, Bucheon St. Mary’s Hospital, The Catholic University of Korea, Seoul 14647, Republic of Korea; 21400254@cmcnu.or.kr; 2 Department of Neurosurgery, Eunpyeong St. Mary’s Hospital, The Catholic University of Korea, Seoul 03312, Republic of Korea; jatagi15@catholic.ac.kr

**Keywords:** radiation exposure, minimally invasive surgery, cervical spine, fluoroscopy, dose reduction, occupational radiation, radiation safety

## Abstract

*Background and Objectives*: Minimally invasive cervical spine surgery (MIS-CSS) relies heavily on intraoperative fluoroscopic imaging, raising concerns about radiation exposure to patients and surgical staff. Unlike lumbar MIS, cervical-specific radiation exposure has not been systematically reviewed, despite distinct anatomical considerations, including proximity to the thyroid gland and lens of the eye. This review aims to quantify intraoperative radiation exposure during MIS cervical spine procedures and evaluate available dose-reduction strategies. *Materials and Methods*: A systematic literature search was conducted across PubMed/MEDLINE, Scopus, and Google Scholar in April 2026 following PRISMA 2020 guidelines. Studies reporting original quantitative radiation data during minimally invasive cervical spine procedures in adult patients (≥10 patients) were included. Quality was assessed using the MINORS tool and the JBI checklist. *Results*: Seven studies encompassing 380 patients were included. Procedures comprised ACDF (four studies), minimally invasive posterior cervical laminoforaminotomy (two studies), and CT-navigated cervical instrumentation (one study). Patient effective doses during ACDF ranged from 0.015 to 1.3 mSv, with thyroid doses of 0.194–0.290 mGy. Standalone ACDF reduced patient dose by 36–58% compared to plated ACDF (*p* < 0.001). Navigation-assisted posterior cervical foraminotomy achieved a median fluoroscopy time of 10 s with negligible staff exposure. Surgeon per-procedure exposure during cervical discectomy (chest 0.122 µSv, lens 3.1 µSv, hands 7.1 µSv) was approximately half that of lumbar discectomy. *Conclusions*: Radiation doses during individual MIS cervical procedures appear to be within occupational safety limits, though the current evidence is insufficient to establish definitive dose thresholds. Standalone implant designs and intraoperative navigation represent effective, complementary dose-reduction strategies. Standardized prospective research is needed to establish cervical-specific radiation safety benchmarks.

## 1. Introduction

Minimally invasive spine surgery (MIS) has transformed the management of cervical spine pathologies over the past two decades, offering significant advantages, including reduced blood loss, decreased postoperative pain, shorter hospital stays, and faster functional recovery compared to traditional open approaches [1,2]. Cervical spine procedures amenable to minimally invasive techniques include anterior cervical discectomy and fusion (ACDF), posterior cervical foraminotomy (MI-PCF), endoscopic cervical discectomy, and percutaneous posterior cervical instrumentation [3,4].

However, a fundamental trade-off inherent to MIS techniques is their increased dependence on intraoperative imaging for anatomical localization, instrument trajectory planning, and hardware placement verification [5,6]. Fluoroscopic guidance using C-arm or O-arm systems remains the cornerstone of intraoperative imaging in MIS, exposing both the patient and the surgical team to ionizing radiation. The World Health Organization has classified ionizing radiation as a known carcinogen, and chronic low-dose occupational exposure has been linked to increased risks of thyroid cancer, cataracts, hematologic malignancies, and skin erythema [7,8].

The as low as reasonably achievable (ALARA) principle is the widely accepted standard for managing occupational radiation exposure. Several strategies have been developed to mitigate radiation exposure during spinal procedures, including lead shielding, surgeon positioning optimization, pulse fluoroscopy, intraoperative navigation, and, more recently, robotic-assisted systems [5,9,10]. While these strategies have been evaluated predominantly in the context of lumbar MIS procedures, the cervical spine presents unique considerations, including proximity to radiosensitive structures (thyroid gland, lens of the eye), different anatomical access corridors, and distinct procedural demands [11,12].

Several systematic reviews have examined radiation exposure in MIS spine surgery broadly, with a predominant focus on lumbar and thoracolumbar procedures [1,2,5]. To our knowledge, no systematic review has specifically addressed radiation exposure during minimally invasive cervical spine procedures. This gap is clinically significant, given the anatomical and procedural differences between cervical and lumbar MIS, and the unique radiation safety considerations of the cervical region.

The purpose of this systematic review is to (1) quantify reported intraoperative radiation exposure during MIS cervical spine procedures; (2) compare radiation exposure between MIS and open cervical approaches where available; (3) evaluate the efficacy of dose-reduction strategies specific to cervical MIS; and (4) identify gaps in the current literature to guide future research.

## 2. Materials and Methods

### 2.1. Study Design and Registration

This systematic review was conducted in accordance with the Preferred Reporting Items for Systematic Reviews and Meta-Analyses (PRISMA) 2020 guidelines (PRISMA checklist is provided as Supplementary Material) [13]. A formal protocol was developed a priori but was not registered in a public registry. To enhance methodological transparency and reproducibility, the Section 2 provides detailed information regarding the search databases, search strategy, eligibility criteria, Rayyan-based study screening process, dual-reviewer study selection, and predefined data extraction variables.

### 2.2. Eligibility Criteria

Studies were included if they (1) reported original data on intraoperative radiation exposure (dose and/or fluoroscopy time) during minimally invasive cervical spine surgical procedures; (2) involved adult patients (aged ≥18 years); (3) were published in English; and (4) were designed as randomized controlled trials, prospective or retrospective cohort studies, case–control studies, or case series with ≥10 patients. Studies exclusively reporting on lumbar or thoracolumbar procedures without cervical-specific data, cadaveric or phantom studies, case reports, conference abstracts, editorials, and narrative reviews were excluded.

### 2.3. Information Sources and Search Strategy

A comprehensive literature search was conducted across PubMed/MEDLINE, Scopus, and Google Scholar in April 2026. No date restrictions were applied. The search strategy combined terms related to three key domains: minimally invasive surgical approach, cervical spine anatomy and procedures, and radiation exposure and imaging.

The PubMed search string was as follows: (“minimally invasive” OR “MIS” OR “percutaneous” OR “endoscopic” OR “tubular” OR “keyhole”) AND (“cervical spine” OR “cervical vertebra” OR “cervical disc” OR “ACDF” OR “anterior cervical” OR “posterior cervical foraminotomy” OR “cervical laminoforaminotomy” OR “cervical pedicle screw”) AND (“radiation” OR “radiation exposure” OR “radiation dose” OR “fluoroscopy” OR “fluoroscopic” OR “ionizing radiation” OR “intraoperative imaging” OR “C-arm” OR “O-arm” OR “navigation” OR “image-guided”). An equivalent search strategy was applied in Scopus using the TITLE-ABS-KEY field tag: TITLE-ABS-KEY ((“minimally invasive” OR “MIS” OR “percutaneous” OR “endoscopic” OR “tubular” OR “keyhole”) AND (“cervical spine” OR “cervical vertebra” OR “cervical disc” OR “ACDF” OR “anterior cervical” OR “posterior cervical foraminotomy” OR “cervical laminoforaminotomy” OR “cervical pedicle screw”) AND (“radiation” OR “radiation exposure” OR “radiation dose” OR “fluoroscopy” OR “fluoroscopic” OR “ionizing radiation” OR “intraoperative imaging” OR “C-arm” OR “O-arm” OR “navigation” OR “image-guided”)). Google Scholar was searched using a simplified string due to platform limitations on Boolean complexity: “minimally invasive cervical spine surgery” AND (“radiation exposure” OR “fluoroscopy” OR “radiation dose”). The first 200 results (20 pages) were screened by title and abstract for relevance, consistent with established systematic review methodology for Google Scholar searches. Reference lists of all included studies and relevant systematic reviews were additionally hand-searched.

### 2.4. Study Selection Criteria

All identified records were imported into Rayyan (Rayyan Systems Inc., Cambridge, MA, USA; https://www.rayyan.ai; accessed on 1 April 2026), a web-based systematic review platform, for deduplication and screening. Title and abstract screening was independently performed by two authors (D.H.K. and J.-T.H.) against predefined eligibility criteria, followed by full-text review of potentially eligible studies. Reasons for exclusion at the full-text stage were documented. Disagreements were resolved through discussion with the corresponding author (J.-W.H.).

### 2.5. Data Extraction

Data were extracted into a standardized spreadsheet by the first author (D.H.K.) and verified by the corresponding author (J.-W.H.). Extracted variables included: study characteristics (first author, year, country, study design, sample size), patient demographics, surgical procedure type and approach, imaging modality, radiation outcomes (dose to surgeon and/or patient, fluoroscopy time, dose–area product), dose-reduction strategies employed, and relevant clinical outcomes (screw accuracy, complication rate).

### 2.6. Methodological Quality Assessment

Methodological quality was assessed using the Methodological Index for Non-Randomized Studies (MINORS) for comparative studies [14] and the Joanna Briggs Institute (JBI) Critical Appraisal Checklist for case series. Each study was rated as having low, moderate, or high risk of bias.

### 2.7. Data Synthesis

Due to anticipated heterogeneity in surgical procedures, imaging modalities, and outcome reporting, a qualitative (narrative) synthesis was the primary analytical approach. Studies were grouped by procedure type, imaging modality, and radiation recipient.

## 3. Results

### 3.1. Study Selection Results

**Figure 1:**
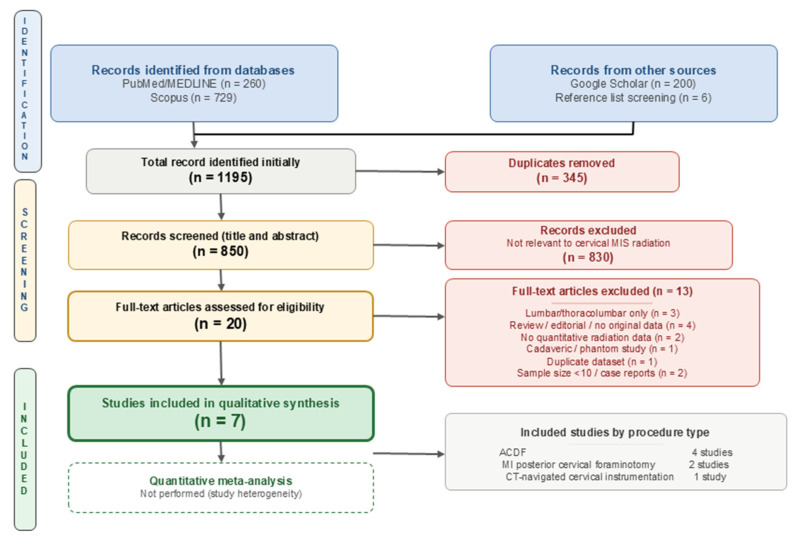
PRISMA 2020 flow diagram illustrating the study selection process.

The systematic search identified a total of 1195 records: 260 from PubMed/MEDLINE, 729 from Scopus, 200 from Google Scholar, and 6 from reference list screening. After the removal of 345 duplicate records, 850 unique records remained for title and abstract screening. Following screening, 830 records were excluded as not relevant, leaving 20 full-text articles assessed for eligibility. Thirteen articles were excluded for the following reasons: exclusively lumbar or thoracolumbar data (*n* = 3), narrative review or editorial without original data (*n* = 4), no quantitative radiation measurements (*n* = 2), cadaveric study design (*n* = 1), duplicate dataset (*n* = 1), and sample size below inclusion threshold (*n* < 10; case reports/series; *n* = 2). Seven studies were included in the final qualitative synthesis (Figure 1).

### 3.2. Study Characteristics

The seven included studies were published between 2016 and 2024 and originated from five countries: the United States (*n* = 2), France (*n* = 1), Greece (*n* = 2), China (*n* = 1), and Canada (*n* = 1). Study designs included prospective studies (*n* = 2, including one multicenter) and retrospective cohort or comparative studies (*n* = 5). The total number of patients across all studies was 380. The procedures evaluated included anterior cervical discectomy and fusion (ACDF; four studies), minimally invasive posterior cervical laminoforaminotomy (MI-PCLF; two studies), and CT-navigated cervical instrumentation with cervical subgroup analysis (one study). Detailed study characteristics are presented in Table 1.

### 3.3. Quality Assessment Results

Domain-level quality assessment was performed using the MINORS tool for comparative studies and the JBI Critical Appraisal Checklist for single-arm case series; results are presented in Supplementary Table S1. One study was rated as high quality (Grelat et al. [19], prospective multicenter design with standardized dosimetry protocols across four centers). The remaining six studies were rated as moderate quality, with no study reporting strategies to address confounding, and prospective data collection was limited to two studies.

### 3.4. Radiation Exposure by Procedure Type

#### 3.4.1. Anterior Cervical Discectomy and Fusion (ACDF)

Four studies reported radiation exposure during ACDF procedures. Metaxas et al. (2017) [17] prospectively measured patient dose in 33 fluoroscopically guided procedures, reporting a mean entrance skin dose of 1.95 mGy, an effective dose of 0.015 mSv, and a mean thyroid absorbed dose of 0.194 mGy. The dose was comparable to a single lateral cervical spine radiograph. In a subsequent computational dosimetry study, Metaxas et al. (2024) [18] evaluated organ doses in 50 ACDF procedures using the VirtualDose™IR software (Virtual Phantoms Inc., Albany, NY, USA; https://www.virtualphantoms.com/our-products/virtualdoseir/; accessed on 1 April 2026), finding a mean thyroid dose of 0.290 mGy. Fusions at the C6/C7 level resulted in significantly higher thyroid and esophageal doses compared to more rostral levels. Chin et al. (2018) [16] compared standalone ACDF (S-ACDF, *n* = 48) with plated ACDF (*n* = 49), demonstrating that S-ACDF reduced patient radiation dose by 38% overall (0.8 ± 0.3 mSv vs. 1.3 ± 0.2 mSv, *p* < 0.001). For single-level procedures, the reduction was 58% (0.5 ± 0.1 mSv vs. 1.2 ± 0.2 mSv, *p* = 0.001).

#### 3.4.2. Minimally Invasive Posterior Cervical Foraminotomy

Vaishnav and Qureshi et al. (2022) [15] evaluated MI-PCLF performed with skin-anchored intraoperative 3D navigation in 21 patients (36 levels). The median patient radiation dose was 2.5 mGy (IQR 1.8–4.9) with a median fluoroscopy time of 10 s (IQR 9–11), almost entirely attributable to the navigation image acquisition. Operating room personnel exposure was negligible because staff stood behind protective shielding during the scan. There were no wrong-level surgeries. Zhong et al. (2022) [20] reported on 34 patients undergoing full-endoscopic posterior cervical foraminotomy with a novel K-wire V-point localization technique, achieving a mean radiation dose of 1.68 ± 0.36 mSv with a short positioning time of 10.68 ± 5.42 min.

#### 3.4.3. CT-Navigated Cervical Instrumentation

**Figure 2:**
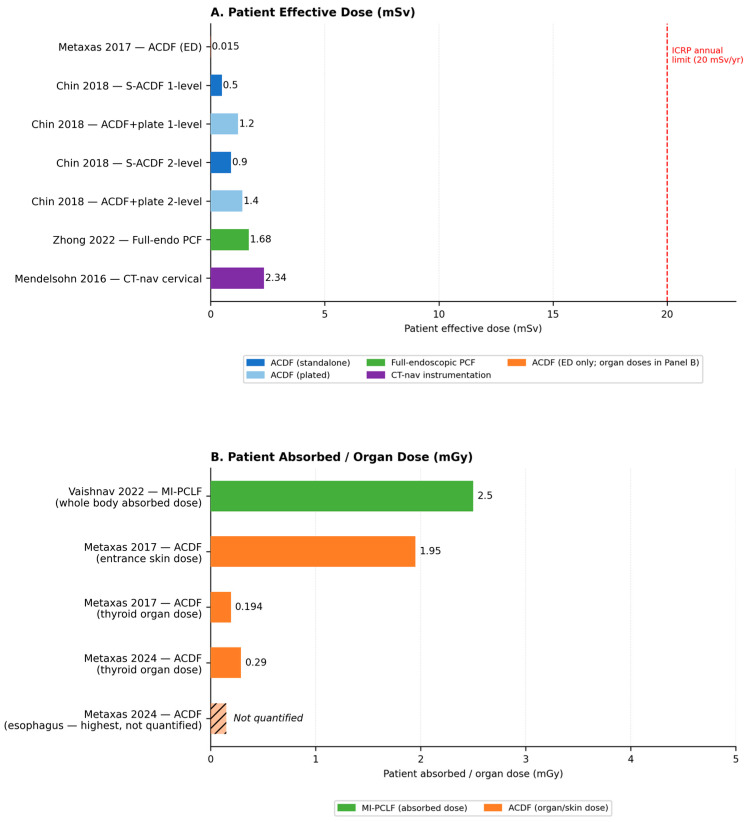
Patient radiation exposure across included studies, stratified by dose metric and unit of measurement [15,16,17,18,20,21]. ACDF, anterior cervical discectomy and fusion; S-ACDF, standalone anterior cervical discectomy and fusion; ED, effective dose; PCF, posterior cervical foraminotomy; MI-PCLF, minimally invasive posterior cervical laminoforaminotomy; CT-nav, computed tomography-based navigation; mSv, millisievert; mGy, milligray; ICRP, International Commission on Radiological Protection.

Mendelsohn et al. (2016) [21] retrospectively analyzed 73 CT-navigated spinal instrumentation cases. The cervical subgroup showed a mean effective dose of 2.34 mSv, 66% lower than thoracolumbar cases (6.93 mSv). Staff radiation dose was 2.5-fold lower with navigation than with conventional fluoroscopy, demonstrating a significant protective benefit for operating room personnel. Patient radiation doses across all procedure types are summarized in Figure 2 (Panel A: effective dose; Panel B: absorbed and organ doses).

### 3.5. Surgeon and Operating Room Staff Radiation Exposure

**Figure 3:**
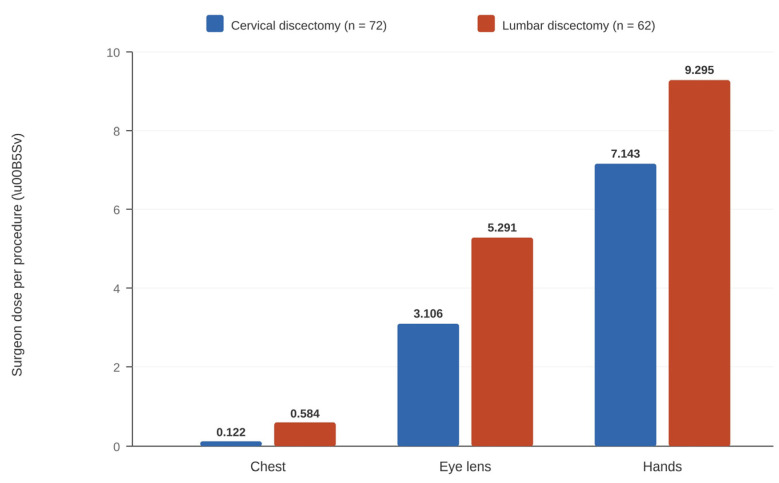
Surgeon radiation exposure per procedure: cervical vs. lumbar discectomy. Data from Grelat et al. 2016 [19], a prospective multicenter study (4 centers, France).

Grelat et al. (2016) [19] provided the most comprehensive surgeon dosimetry data in a prospective multicenter study of 72 cervical discectomies across four centers in France. Per-procedure surgeon exposure averaged 0.122 µSv at the chest, 3.106 µSv at the lens of the eye, and 7.143 µSv at the hands. The dose–area product was 35.7 ± 72.1 cGy·cm^2^, with a fluoroscopy time of 19.7 ± 13.7 s. Surgeon exposure during cervical procedures was consistently lower than during lumbar discectomy in the same cohort (chest: 0.584 µSv, lens: 5.291 µSv, hands: 9.295 µSv; Figure 3).

### 3.6. Dose-Reduction Strategies

Three categories of dose-reduction strategies were identified (Table 2). First, in terms of implant design modification, standalone cervical cages eliminated the need for plate and screw fluoroscopy, reducing patient dose by 36–58% [16]. Second, regarding intraoperative navigation, skin-anchored 3D navigation achieved fluoroscopy times as low as 10 s with negligible staff exposure [15], and CT-based navigation reduced staff exposure 2.5-fold versus conventional fluoroscopy [21]. Lastly, optimized surgical localization techniques (V-point K-wire method) also demonstrated low radiation doses without requiring advanced equipment [20].

## 4. Discussion

This systematic review provides the first comprehensive synthesis of the literature specifically addressing radiation exposure during minimally invasive cervical spine procedures. Unlike previous systematic reviews on radiation exposure in MIS, which primarily focused on lumbar and thoracolumbar procedures or generalized occupational radiation reduction strategies [1,2,5], the present review focused specifically on MIS-CSS. In addition to summarizing overall radiation doses, this review highlights cervical-specific considerations, including radiation exposure to radiosensitive structures such as the thyroid gland and lens of the eye, differences in fluoroscopic localization workflows, and procedure-specific dose-reduction strategies for cervical procedures such as ACDF and posterior cervical foraminotomy. These distinctions suggest that radiation exposure patterns and safety considerations in cervical MIS may not be directly extrapolated from the lumbar MIS literature.

### 4.1. Summary of Key Findings

Patient radiation doses across all cervical MIS procedures ranged from 0.015 mSv (ACDF effective dose) to 2.34 mSv (CT-navigated cervical instrumentation). These per-procedure values are substantially lower than a single routine lumbar spine CT scan (~7.5 mSv) [21] and fall well below the annual occupational limits established by the International Commission on Radiological Protection (ICRP; 20 mSv/year) and the National Council on Radiation Protection and Measurements (NCRP; 50 mSv/year) [7]. It should be noted, however, that these occupational limits apply specifically to radiation workers and are not directly applicable to patient exposures. These findings should also be interpreted with caution, given the limited number of studies and heterogeneous dosimetry methods, and cumulative annual exposure—rather than individual procedure dose—remains the more clinically relevant metric for the occupational safety of surgical staff.

### 4.2. Comparison with Lumbar MIS Literature

Several included studies reported relatively low radiation exposure during cervical MIS procedures compared with previously published lumbar MIS literature. Grelat et al. [19] directly demonstrated that surgeon radiation exposure during cervical discectomy was approximately half that of lumbar discectomy within the same cohort. Mendelsohn et al. [21] showed that the patient’s effective dose for cervical CT-navigated instrumentation was 66% lower than for thoracolumbar cases. These differences likely reflect the lower tissue thickness in the cervical region, requiring less radiation for adequate imaging, fewer instrumented levels, and shorter fluoroscopy times needed for level localization in anterior cervical approaches.

### 4.3. Heterogeneity of Radiation Metrics and Reporting

Interpretation of radiation exposure across studies requires caution because the included studies reported heterogeneous radiation metrics, including absorbed dose (mGy), effective dose (mSv), fluoroscopy time, and dose–area product. These parameters reflect different aspects of radiation exposure and are influenced by variations in dosimetry methodology, imaging protocols, dosimeter placement, patient anatomy, and procedural workflow. Consequently, direct quantitative comparison between studies remains limited, and the findings of the present review should be interpreted primarily in a qualitative and descriptive context.

### 4.4. Role of Navigation and Emerging Technologies

Several included studies suggested that intraoperative navigation may help reduce fluoroscopy time and staff radiation exposure. Skin-anchored 3D navigation for MI-PCLF reduced fluoroscopy time to just 10 s with negligible staff exposure [15], while CT-based navigation reduced staff exposure by 2.5-fold versus conventional fluoroscopy [21].

### 4.5. Clinical Implications

Several preliminary observations from this review may inform clinical practice. For ACDF procedures, standalone cage designs without anterior plating offer a 36–58% dose reduction without requiring additional equipment, suggesting this may represent a practical dose-reduction option [16]. For posterior cervical procedures, intraoperative navigation may be considered when available and feasible, as data suggest it can reduce fluoroscopy time substantially. Regardless of technology, adherence to ALARA principles remains fundamental: thyroid shielding is particularly important given the proximity of the thyroid gland to the cervical surgical field, appropriate C-arm positioning should be ensured, and pulsed rather than continuous fluoroscopy should be used when possible [7,8].

### 4.6. Limitations

This review has several important limitations. First, the small screening team (two reviewers) without external adjudication may increase the risk of selection bias, although disagreements were resolved through consensus with the corresponding author. Second, the English-language restriction may have excluded relevant studies published in other languages, particularly from Asian centers with high volumes of endoscopic cervical surgery. Third, the review protocol was not prospectively registered in PROSPERO, which may limit methodological transparency. However, the review methodology, eligibility criteria, and study selection process were predefined and are described in detail in accordance with PRISMA 2020 recommendations. Fourth, significant heterogeneity in radiation measurement methods (entrance skin dose vs. effective dose vs. organ dose), dosimeter placement, and outcome reporting precluded quantitative meta-analysis. Fifth, publication bias cannot be excluded. Finally, the overall small number of cervical-specific studies (*n* = 7) limits the strength and generalizability of our conclusions. The predominance of retrospective single-center studies with relatively small cohort sizes and heterogeneous radiation reporting methodologies, with only one high-quality prospective multicenter study identified, further constrains the evidence base.

### 4.7. Future Directions

This review identifies several critical research gaps. Prospective studies using standardized dosimetry protocols across multiple cervical MIS procedures are urgently needed to enable quantitative comparison and meta-analysis. The role of augmented reality navigation, artificial intelligence-based dose optimization, and ultra-low-dose imaging protocols warrants investigation in the cervical MIS context. Comparative studies between navigation-assisted and conventional fluoroscopic techniques for cervical procedures are notably absent from the literature. Long-term cohort studies tracking health outcomes in high-volume cervical spine surgeons would provide critical data for occupational safety guidelines. Greater attention should also be directed toward radiation exposure to non-surgical operating room staff.

## 5. Conclusions

This systematic review, the first to specifically address radiation exposure during minimally invasive cervical spine procedures, suggests that individual procedure doses may be within occupational safety limits based on the available evidence. Navigation-assisted techniques show promise for reducing surgeon and staff radiation exposure, while standalone implant designs offer a practical, equipment-free dose-reduction strategy. The evidence base remains limited by small sample sizes, heterogeneous dosimetry methods, and the absence of prospective comparative studies. Standardized, multicenter prospective research is urgently needed to establish evidence-based radiation safety guidelines specific to cervical MIS procedures.

## Figures and Tables

**Table 1:** Characteristics of included studies.

Author (Year)	Country	Design	N	Procedure	Imaging Modality	Patient Dose (Value, Unit)	Surgeon/Staff Dose (Value, Unit)	Measurement Method	Anatomical Site	FT (s)
Vaishnav et al. (2022) [15]	USA	Retro	21	MI-PCLF	Skin-anchored 3D ION (C-arm)	2.5 mGy (median; IQR 1.8–4.9)	Negligible (staff behind lead shield during ION acquisition)	C-arm dosimetric readout	Whole body (patient); OR personnel (staff)	10
Chin et al. (2018) [16]	USA	Retro comp.	97	S-ACDF vs. ACDF + plate	C-arm fluoro	S-ACDF: 0.8 ± 0.3 mSv Plate: 1.3 ± 0.2 mSv	NR	Fluoroscopy machine readout (formula- based calculation)	Whole body (patient)	NR
Metaxas et al. (2017) [17]	Greece	Prospective	33	ACDF	C-arm fluoro	ESD: 1.95 mGy ED: 0.015 mSv Thyroid: 0.194 mGy	NR	KAP meter + MC simulation (CALDoseX software)	Cervical skin; Whole body; Thyroid	NR
Metaxas et al. (2024) [18]	Greece	Retro	50	ACDF	C-arm + VirtualDose-IR	Thyroid: 0.290 mGy Esophagus: highest OD	NR	KAP meter + MC simulation (VirtualDose-IR software)	Thyroid; Esophagus; Salivary glands	NR
Grelat et al. (2016) [19]	France	Prosp. MC	72	Cervical discectomy (ACDF/ACDA)	C-arm fluoro	DAP: 35.7 ± 72.1 cGy·cm^2^	Chest: 0.122 µSv Lens: 3.106 µSv Hands: 7.143 µSv	C-arm DAP meter (patient); EPD + TLD dosimeters (surgeon)	Whole body (patient); Chest, Lens, Hands (surgeon)	19.7
Zhong et al. (2022) [20]	China	Retro	34	Full-endo PCF (V-point K-wire technique)	C-arm fluoro (A/P view)	1.68 ± 0.36 mSv	NR	Fluoroscopy machine readout	Whole body (patient)	NR
Mendelsohn et al. (2016) [21]	Canada	Retro cohort	73	CT-navigated spinal instrumentation (cervical subgroup)	iCT navigation (O-arm)	Cervical: 2.34 mSv T/L: 6.93 mSv	2.5× lower vs. conventional fluoro (surrogate measure)	DAP + DLP conversion to effective dose (NCRP/AAPM conversion factors)	Whole body (patient); Whole body (staff, surrogate)	NR

MI-PCLF, minimally invasive posterior cervical laminoforaminotomy; S-ACDF, standalone ACDF; PCF, posterior cervical foraminotomy; ION, intraoperative navigation; iCT, intraoperative CT; ACDA, artificial cervical disc arthroplasty; FT, fluoroscopy time; NR, not reported; Retro, retrospective; Prosp. MC, prospective multicenter; T/L, thoracolumbar; IQR, interquartile range; ESD, entrance skin dose; ED, effective dose; OD, organ dose; DAP, dose–area product; DLP, dose–length product; KAP, kerma–area product; MC, Monte Carlo; EPD, electronic personal dosimeter; TLD, thermoluminescent dosimeter; mGy, milligray; mSv, millisievert; µSv, microsievert; A/P, anteroposterior.

**Table 2:** Summary of dose-reduction strategies evaluated in included studies.

Study	Procedure	Strategy	Control Dose	Intervention Dose	*p*	Reduction
Chin [16]	ACDF 1-level	Standalone cage	1.2 ± 0.2 mSv	0.5 ± 0.1 mSv	<0.001	58%
Chin [16]	ACDF 2-level	Standalone cage	1.4 ± 0.3 mSv	0.9 ± 0.2 mSv	<0.001	36%
Vaishnav [15]	MI-PCLF	Skin-anchored 3D ION	N/A	2.5 mGy; FT 10 s	—	Staff ≈ 0
Mendelsohn [21]	Cerv. instr.	iCT navigation	Fluoro-guided	Cerv. 2.34 mSv	NR	Staff 2.5× ↓
Zhong [20]	Endo PCF	V-point K-wire	Conventional fluoro	1.68 ± 0.36 mSv	NR	Low dose

Cerv. instr., cervical instrumentation; Cerv., cervical spine cases; N/A, not applicable; ↓, reduction in radiation exposure.

## Data Availability

All data generated or analyzed during this study are included in this published article and its Supplementary Materials. The search strategies and extracted data are available from the corresponding author upon reasonable request.

## Supplementary Materials

The following supporting information can be downloaded at: https://www.mdpi.com/article/10.3390/medicina62050977/s1. PRISMA Checklist; Table S1: Risk-of-Bias Assessment: MINORS and JBI Domain-Level Quality Evaluation.

## Abbreviations

The following abbreviations are used in this manuscript:

ACDF	Anterior cervical discectomy and fusion
ALARA	As low as reasonably achievable
CT	Computed tomography
ED	Effective dose
ESD	Entrance skin dose
FT	Fluoroscopy time
ICRP	International Commission on Radiological Protection
iCT	Intraoperative computed tomography
ION	Intraoperative navigation
IQR	Interquartile range
JBI	Joanna Briggs Institute
MI-PCF	Minimally invasive posterior cervical foraminotomy
MI-PCLF	Minimally invasive posterior cervical laminoforaminotomy
MIS	Minimally invasive surgery
MIS-CSS	Minimally invasive cervical spine surgery
MINORS	Methodological Index for Non-Randomized Studies
NCRP	National Council on Radiation Protection
NR	Not reported
OD	Organ dose
PCF	Posterior cervical foraminotomy
PRISMA	Preferred Reporting Items for Systematic Reviews and Meta-Analyses
S-ACDF	Standalone anterior cervical discectomy and fusion
